# NRF2 pathway activation predicts poor prognosis in lung cancer: a cautionary note on antioxidant interventions

**DOI:** 10.1007/s11357-025-01736-0

**Published:** 2025-06-19

**Authors:** Zoltan Ungvari, Otília Menyhart, Andrea Lehoczki, Monika Fekete, Vince Fazekas-Pongor, Alberto Ocana, Peter Varga, Balázs Győrffy

**Affiliations:** 1 Vascular Cognitive Impairment, Neurodegeneration and Healthy Brain Aging Program, Department of Neurosurgery, University of Oklahoma Health Sciences Center, Oklahoma City, OK USA; 2 Stephenson Cancer Center, University of Oklahoma, Oklahoma City, OK USA; 3 Oklahoma Center for Geroscience and Healthy Brain Aging, University of Oklahoma Health Sciences Center, Oklahoma City, OK USA; 4 Department of Health Promotion Sciences, College of Public Health, University of Oklahoma Health Sciences Center, Oklahoma City, OK USA; 5 International Training Program in Geroscience, Doctoral College/Institute of Preventive Medicine and Public Health, Semmelweis University, Budapest, Hungary; 6 Cancer Biomarker Research Group, Institute of Molecular Life Sciences, Hungarian Research Network, Magyar Tudósok Körútja 2, Budapest, 1117 Hungary; 7 National Laboratory for Drug Research and Development, Magyar Tudósok Körútja 2, Budapest, 1117 Hungary; 8 Department of Bioinformatics, Semmelweis University, Budapest, H-1094 Hungary; 9 Doctoral College, Health Sciences Division, Semmelweis University, Budapest, Hungary; 10 Institute of Preventive Medicine and Public Health, Semmelweis University, Budapest, Hungary; 11 Fodor Center for Prevention and Healthy Aging, Semmelweis University, Budapest, Hungary; 12 Experimental Therapeutics in Cancer Unit, Instituto de Investigación Sanitaria San Carlos (IdISSC), and CIBERONC, Madrid, Spain; 13 INTHEOS-CEU-START Laboratory, Facultad de Medicina, Universidad CEU San Pablo, 28668 Boadilla del Monte, Madrid, Spain; 14 Department of Biophysics, Medical School, University of Pecs, Pecs, H-7624 Hungary

**Keywords:** Lung cancer, Non-small cell lung cancer, Prognosis, Aging, Stress resilience, Oxidative stress, Antioxidants, Resveratrol, Nutraceuticals, KEAP1, Redox biology, Cancer biomarkers, Gene expression signature, Aging-related disease, Redox signaling, Dietary supplements, Transcriptomics, Prognostic biomarkers, Cancer prevention, Systems biology, Therapy resistance

## Abstract

Lung cancer is a leading cause of cancer-related mortality worldwide. As an age-related disease, its pathogenesis is shaped by several molecular hallmarks of aging, including impaired DNA repair and diminished stress resilience. The transcription factor NRF2 (nuclear factor erythroid 2–related factor 2) is a master regulator of oxidative stress defense and cellular survival. While NRF2 activation is protective in aging tissues, it may also be exploited by cancer cells to promote tumor progression and therapy resistance. This study aims to evaluate whether NRF2 pathway activation predicts clinical outcomes in lung cancer, with potential implications for the use of NRF2-inducing compounds. We analyzed transcriptomic and survival data from 2167 lung cancer patients using the KM Plotter database. A validated 14-gene NRF2 activation signature was used to stratify tumors by NRF2 pathway activity. Associations with overall survival (OS), first progression (FP), and post-progression survival (PPS) were assessed using Cox regression models and Kaplan–Meier analysis. High NRF2 signature expression was significantly associated with poorer OS (HR = 1.59, *p* = 1.3E−9), FP (HR = 1.61, *p* = 2.6E−5), and PPS (HR = 1.6, *p* = 0.002). The negative prognostic effect was most pronounced in patients with adenocarcinoma, node-negative disease, and in female patients. These findings highlight the dual role of NRF2 in promoting stress resilience and enabling cancer cell survival. NRF2 activation is a predictor of poor clinical outcomes in lung cancer. Given the widespread use of NRF2-inducing compounds such as resveratrol and sulforaphane, these findings raise important concerns about their safety in individuals at risk for or living with cancer. Our results underscore the importance of context-specific evaluation of NRF2-targeted interventions and caution against the indiscriminate use of NRF2-activating agents in aging populations, particularly in individuals at risk for lung cancer.

## Introduction

Lung cancer remains the leading cause of cancer-related death globally, accounting for approximately 1.8 million deaths each year [1]. Despite improvements in early detection and the advent of targeted therapies and immunotherapy, prognosis remains poor, especially in advanced stages [1, 2]. Non-small cell lung cancer (NSCLC), which includes adenocarcinoma and squamous cell carcinoma, comprises the majority of lung cancer cases and is frequently diagnosed in older adults [1, 3, 4].

Cancer is widely recognized as an age-related disease [5]. Age is the strongest risk factor for most cancers, and many hallmarks of aging—such as genomic instability, epigenetic alterations, mitochondrial dysfunction, and cellular senescence—are also fundamental to cancer biology [5–9]. One key feature of aging is the progressive decline in the ability of cells and tissues to respond to oxidative stress [9–13]. In contrast, cancer cells often evolve mechanisms to enhance stress resistance [14, 15], enabling them to thrive in hostile microenvironments, evade therapy, and metastasize [16–27].

A central regulator of cellular stress resilience is NRF2 (nuclear factor erythroid 2–related factor 2), a transcription factor that orchestrates the expression of genes involved in antioxidant defense, detoxification, DNA repair, proteostasis, and metabolic reprogramming [16, 18–21, 24]. While NRF2 activation is generally considered beneficial in the context of aging and chronic disease—promoting cytoprotection and tissue homeostasis—it may also be hijacked by cancer cells to support survival, proliferation, and treatment resistance [15, 16, 18–21, 24].

In this study, we explored the prognostic impact of NRF2 pathway activation in lung cancer. We used a validated 14-gene expression signature reflective of NRF2 activation and leveraged the KM Plotter database, which links transcriptomic profiles to clinical outcomes. Specifically, we investigated how NRF2 pathway activation correlates with overall survival (OS), time to first progression (FP), and post-progression survival (PPS) across a large cohort of lung cancer patients, including histological and clinical subgroups.

## Methods

### Database construction and data preprocessing

Microarray data were retrieved from the Gene Expression Omnibus (GEO; http://www.ncbi.nlm.nih.gov/geo/) to assemble a robust dataset linking gene expression with clinical outcomes in lung cancer [28]. Inclusion criteria were (i) availability of raw microarray files, (ii) documented overall survival (OS) or first progression (FP) time, and (iii) a minimum sample size of 30 patients. Post-progression survival (PPS) time was defined as the time length between first progression and overall survival. Only datasets generated on Affymetrix HG-U133A (GPL96) and HG-U133 Plus 2.0 (GPL570) platforms were included due to their widespread use and the shared 22,277 probe sets. Cross-platform harmonization was prioritized to mitigate variability in expression quantification arising from technological differences.

All arrays underwent standardized preprocessing to ensure analytical consistency. Expression values were normalized at the single-array level using the MAS5 algorithm, demonstrating high concordance with RT-PCR measurements [29]. Expression values were globally scaled to a fixed mean of 1000 to reduce batch effects across the 22,277 shared probe sets. Only probes present on GPL96 were retained to eliminate platform-specific bias [30]. The JetSet algorithm was applied to select the most reliable probe set per gene. Technical replicates—samples with identical expression profiles— were collapsed by retaining a single representative. Quality control metrics included background signal, noise, and the percentage of present calls. Experimental integrity was assessed using bioBCD spike-in controls, and RNA quality was evaluated via 3′/5′ signal ratios for *GAPDH* and *ACTB*. Samples failing any QC criterion or falling outside the 95% confidence interval of continuous metrics were excluded. This multi-step pipeline ensured high technical fidelity suitable for downstream survival analyses [31].

### The gene signature of NRF2 pathway activation

To assess the prognostic impact of NRF2 pathway activation in lung cancer, we employed a 14-gene “core NRF2 signature” defined by Luo et al. [32]. Using a multifaceted strategy, the authors compiled seven RNA-sequencing datasets capturing both pharmacological and genetic activation of NRF2 in multiple cancer cell lines and fibroblasts. Differentially upregulated genes were compared across all conditions and then screened against *KEAP1/NFE2L2*-mutated cohorts in TCGA and the Cancer Cell Line Encyclopedia. Subsequent cross-validation in independent human and rodent data yielded a final 14-gene “core NRF2 signature”. Composed of *AKR1C3*, *OSGIN1*, *SLC7A11*, *GCLM*, *SRXN1*, *NQO1*, *GCLC*, *ABHD4*, *PIR*, *GSR*, *FTL*, *ME1*, *FTH1*, and *EPHX1*, the 14-gene panel serves as a surrogate for NRF2 pathway activation across diverse tumor types. We investigated the impact of the “core NFR2 signature”, represented by the weighted mean expression of the 14 genes, on survival outcomes (OS, FP, and PPS) of lung cancer patients.

### Survival analysis

Associations between the NFR2 signature and OS, FP, and PPS were evaluated using Cox proportional hazards models implemented in the R package *survival* (v2.38; http://CRAN.R-project.org/package=survival). Log-rank *p* values, hazard ratios (HR), and 95% confidence intervals (CI) were computed for each gene. All possible cutoffs between the lower and upper quartiles of expression were systematically evaluated, with each tested in an independent Cox model, to avoid arbitrary dichotomization. The cutoff yielding the lowest *p* value was selected for the final Kaplan–Meier curve generation (“best cutoff”) while correcting for multiple testing using the false discovery rate (FDR) method. Findings were considered significant at concurrent *p* < 0.05 and FDR ≤ 20% values.

In the post-progression survival analysis, the best cutoff method produced significant *p* values; however, in subgroup analyses, the corresponding FDRs exceeded 20%, likely reflecting the limited sample size in this patient cohort. To strengthen confidence in our findings in these settings, we also performed Cox regression using a trichotomization approach, separating samples into the lowest and highest expression quartiles. This complementary method helps in reinforcing the potential relevance of the NRF2 gene signature in predicting PPS outcomes.

## Results

### Database construction

A total of 2167 lung cancer cases comprise the gene array database, of which 1406 (64.9%) have both overall survival (OS) data and gene-signature expression, and 870 (40.2%) have first-progression (FP) data (Table 1). Post-progression survival (PPS) data are computed for 242 patients (11.2%). By histologic subtype, adenocarcinomas represent the largest subgroup at about 60% of all cases, followed by squamous cell carcinomas at 43%. In comparison, large cell and large cell neuroendocrine tumors collectively account for less than 6%. Men constitute the majority (71.3%) of the cohort, although fewer than half have FP data, and only 172 have PPS information available; similarly, of the 925 female patients, 294 have FP, and 70 have PPS data. Staging details (T, N, M) are complete in only a subset of cases, with approximately half at T1–T2 and one third at N0; few samples show M1 disease. Grading information was at hand for roughly one quarter of patients but is not linked to survival endpoints or gene expression; therefore, they were excluded from further analyses. Table 1 summarizes these clinical characteristics and OS, FP, and PPS data availability.

**Table 1:** Clinical characteristics of the included samples

Classification		# of patients in the lung cancer database	# of patients with overall survival and gene signature expression	# of patients with first progression and gene signature expression	# of patients with post-progression survival and gene signature expression
*All*		2167	1406	870	242
*Histology*	Adenocarcinoma	1308	670	526	141
	Squamous cell carcinoma	931	526	220	51
	Large cell carcinoma	63	52	10	3
	Large cell neuroendocrine	52	52	44	25
*Sex*	Male	1545	819	576	172
	Female	925	476	294	70
*Grade*	1	189	0	0	0
	2	302	0	0	0
	3	75	0	0	0
*AJCC Stage T*	1	467	220	212	48
	2	672	190	185	72
	3	100	33	29	18
	4	44	21	20	13
*AJCC Stage N*	0	848	324	313	82
	1	296	104	100	46
	2	105	30	27	17
*AJCC Stage M*	0	819	459	442	149
	1	10	8	6	4
*Smoker*	Yes	1007	330	297	96
	No	247	143	141	40

### Prognostic relevance of the NRF2-related gene signature

#### Overall survival

**Fig. 1:**
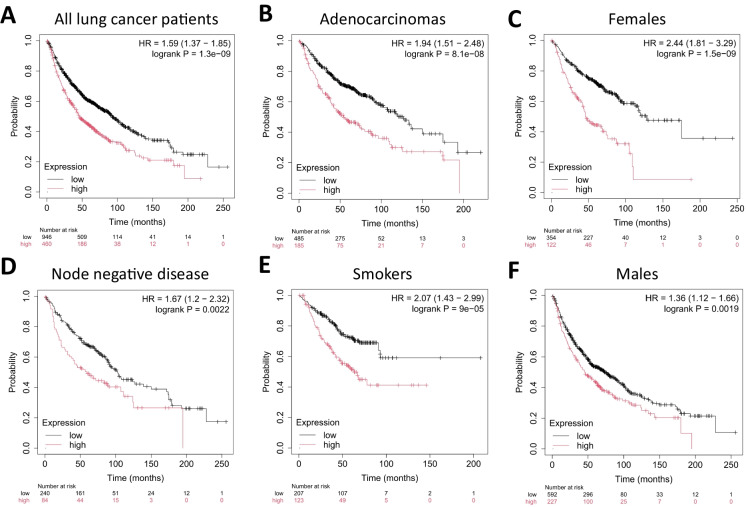
High mean expression of the NRF2-related gene signature correlated with worse overall survival (OS) in lung cancer. The association was significant in **A** all lung cancer patients, **B** patients diagnosed with adenocarcinoma, **C** females, **D** node-negative patients (AJCC Stage N0), **E** smokers, and **F** males. *HR hazard rate*

High expression of the NRF2-related gene signature was consistently associated with significantly worse OS in lung cancer patient cohorts. Across all lung cancer patients, those in the high-expression group showed a hazard ratio (HR) of 1.59 (95% CI 1.37–1.85; *p* = 1.3E−9), reflecting significant prognostic impact (Fig**.** 1A). Subgroup analysis revealed a similarly poor prognosis for adenocarcinomas (HR = 1.94; 95% CI 1.51–2.48; *p* = 8.1E−8, Fig**.** 1B), whereas large cell carcinoma exhibited an HR of 2.25 (95% CI 1.07–4.75; *p* = 0.029) but a higher FDR (~50%), likely owing to smaller sample size. There was no significant association between OS and the expression of the 14-gene signature in lung squamous carcinomas (*p* > 0.1).

Additional subgroup analyses revealed significant survival differences across various clinical categories. Among female patients, high-expression carriers exhibited the most pronounced outcome disparity (HR = 2.44, 95% CI 1.81–3.29; *p* = 1.5E−9, Fig. 1C), whereas a more minor but still significant effect was observed in males (HR = 1.36, 95% CI 1.12–1.66; *p* = 0.0019, Fig. 1F). Notably, patients diagnosed with a node-negative disease (AJCC stage N0) showed poorer prognosis with elevated expression (HR = 1.67, 95% CI 1.2–2.32; *p* = 0.0022, Fig. 1D), suggesting early-stage tumors may already exhibit NRF2-associated risk. Furthermore, smokers with a high expression of the signature experienced markedly worse survival (HR = 2.07, 95% CI 1.43–2.99; *p* = 9E−5, Fig. 1E). Collectively, these data underscore the adverse prognostic influence of the gene signature across diverse clinical and demographic subgroups.

#### First progression

**Fig. 2:**
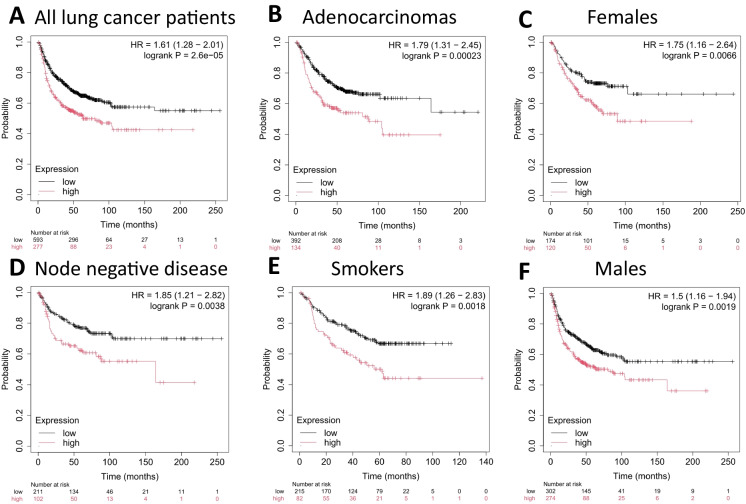
High NRF2 gene signature expression was associated with a shorter time to first progression (FP) in lung cancer. The association was significant in **A** all lung cancer patients, **B** adenocarcinoma patients, **C** females, **D** node-negative patients (AJCC Stage N0), E smokers, and F males. *HR hazard rate*

Analysis of first-progression (FP) outcomes revealed that patients with elevated expression of the NRF2-associated gene set experienced significantly shorter time to progression. High-expression carriers across all lung cancer patients showed an HR of 1.61 (95% CI 1.28–2.01; *p* = 2.6E−5, Fig. 2A). Adenocarcinoma displayed the strongest association (HR = 1.79; 95% CI 1.31–2.45; *p* = 0.00023, Fig. 2B), indicating that this histologic subtype is particularly sensitive to NRF2-driven effects on tumor progression. Even patients with node-negative disease, ostensibly at earlier disease stages, showed increased progression risk (HR = 1.85; 95% CI 1.21–2.82; *p* = 0.0038, Fig. 2D). Furthermore, elevated expression correlated with poorer FP in both females (HR = 1.75; 95% CI 1.16–2.64; *p* = 0.0066, Fig. 2C) and males (HR = 1.50; 95% CI 1.16–1.94; *p* = 0.0019, Fig. 2F). Among smokers, high-expression patients likewise demonstrated substantially worse outcomes (HR = 1.89; 95% CI 1.26–2.83; *p* = 0.0018, Fig. 2E), underscoring that lifestyle risk factors may further accentuate the negative prognostic influence of NRF2-associated gene activation.

#### Post-progression survival

**Fig. 3:**
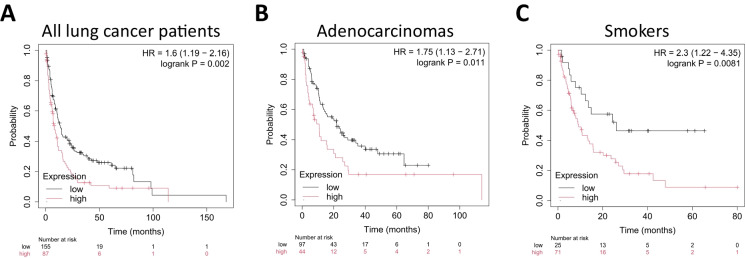
High NRF2 gene signature expression was associated with worse post-progression survival in lung cancer. **A** The association among the entire lung cancer patient cohort with the “best cutoff” method was significant. A similar trend was observed in **B** patients diagnosed with adenocarcinomas and **C** smokers. Additional Cox regressions using a trichotomization approach validated the consistency of subgroup findings. *HR hazard rate*

Among patients with available post-progression survival (PPS) data, high expression of the NRF2-associated gene signature correlated with significantly worse outcomes in the overall lung cancer cohort (HR = 1.6; 95% CI 1.19–2.16; log-rank *p* = 0.002, FDR=20%, Fig. 3A). Subgroup analyses indicated a similar trend in adenocarcinomas (HR = 1.75; 95% CI 1.13–2.71; *p* = 0.011, Fig. 3B) and smokers (HR = 2.3; 95% CI 1.22–4.35; *p* = 0.0081, Fig. 3C). Due to FDR exceeding the 20% cutoff, we conducted an additional Cox regression analysis using a trichotomization approach. Specifically, we stratified patients into three groups (lowest, middle, and highest terciles) based on gene signature expression and then compared survival outcomes between the lowest and highest expression groups. In the overall lung cancer cohort, this method yielded significant differences in post-progression survival (HR = 1.58; 95% CI 1.1–2.25; *p* = 0.012). Similarly, subgroup analyses of adenocarcinoma (HR = 1.94; 95% CI 1.15–3.29; *p* = 0.012) and smokers (HR = 2.03; 95% CI 1.08–3.81; *p* = 0.024) demonstrated consistent risk elevations in the high-expression group. Our findings support the potential role of NRF2 pathway activation in predicting outcomes beyond the first progression and warrant further investigation in larger cohorts.

In summary, our analyses demonstrate that elevated expression of the NRF2-associated gene signature consistently predicts poorer survival outcomes—encompassing overall survival, time to first progression, and post-progression survival—in diverse lung cancer populations. Key subgroups, such as adenocarcinoma, early-stage disease, and smokers, appear primarily influenced by NRF2 pathway activation.

## Discussion

Our study provides compelling evidence that activation of the NRF2 stress response pathway—measured using a validated 14-gene expression signature—is associated with significantly worse clinical outcomes in lung cancer. Across a cohort of more than 2000 patients, high NRF2 signature expression predicted shorter overall survival, accelerated progression, and reduced post-progression survival.

These findings align with the dualistic role of NRF2 in cancer [16, 18–22, 24–26]. NRF2 has been widely recognized as a longevity-associated gene, with evolutionary conservation across species [33]. For instance, activation of the NRF2 ortholog *skn-1* in *Caenorhabditis elegans* extends lifespan and enhances stress resistance [13], while similar effects are observed in *Drosophila* and murine models [34–40]. In normal and aging tissues, NRF2 plays a protective role by maintaining redox homeostasis [41, 42], promoting DNA repair [43], supporting proteostasis, and mitigating cellular damage—mechanisms that collectively delay functional decline and suppress tumorigenesis [33, 44–49].

However, in the context of established malignancy, persistent or aberrant NRF2 activation can be hijacked by cancer cells to promote survival and progression [16, 18–22, 24–26]. Tumor cells leverage NRF2-driven pathways to neutralize oxidative stress, evade immune detection, rewire metabolism, and resist chemotherapy and radiotherapy [16, 18–22, 24–26]. This functional dichotomy—where NRF2 is beneficial in the context of aging but detrimental in cancer—is increasingly recognized in oncology [16, 18–22, 24–26]. Notably, activating mutations in *NFE2L2* or inactivating mutations in its repressor *KEAP1* are frequently observed in lung cancer and are associated with NRF2 hyperactivation, poor prognosis, and treatment resistance [15, 27, 50–62].

Recent evidence further supports the notion that NRF2 plays a multifaceted role in cancer progression across a wide spectrum of malignancies [50–68]. A comprehensive transcriptomic analysis by Luo et al. validated the core 14-gene NRF2 target signature used in our study across diverse datasets and cellular models [32]. Their NRF2 activity score was shown to predict resistance to oxidative stress-inducing drugs and radiotherapy and was strongly associated with adverse prognosis in multiple cancer types, including lung adenocarcinoma, hepatocellular carcinoma, renal clear cell carcinoma, and acute myeloid leukemia [32]. These findings lend independent validation to our results and underscore the importance of NRF2-driven stress resilience in shaping cancer outcomes [50–62]. Interestingly, they also highlight context-specific nuances: in some cancers like endometrial carcinoma, NRF2 activation was linked to improved prognosis, likely due to co-occurring PTEN mutations [32]. This complexity reinforces the need to interpret NRF2 activation within the broader molecular and clinical landscape.

The strongest prognostic effects in our study were observed in patients with lung adenocarcinoma, node-negative disease, and smokers. This suggests that NRF2 activation may already be functionally relevant in early-stage tumors and that tobacco-associated oxidative stress may select for NRF2-driven adaptations. Of note, female patients with high NRF2 expression exhibited the greatest survival disadvantage, an observation that warrants further mechanistic exploration, potentially related to sex-specific differences in redox biology or hormone-mediated regulation. Our results are also consistent with prior studies linking NRF2 pathway activation to aggressive tumor behavior and therapy resistance [50–62]. Importantly, our study extends this knowledge by demonstrating prognostic relevance across multiple survival endpoints and diverse clinical subgroups using a robust, harmonized transcriptomic database.

From a translational perspective, these findings raise the possibility that NRF2 pathway activity could serve as a biomarker for prognosis and perhaps therapeutic stratification. While targeting NRF2 directly remains challenging, inhibitors of downstream metabolic and antioxidant pathways may offer a feasible strategy to disrupt NRF2-driven tumor resilience.

While NRF2 activation is often portrayed as universally beneficial, particularly in the context of aging and chronic disease prevention, our findings—and those of others—underscore the potential dangers of indiscriminate or prolonged NRF2 stimulation, particularly in individuals with undiagnosed or early-stage malignancies. Numerous over-the-counter compounds and dietary supplements, including resveratrol [69–71], sulforaphane [72], curcumin [73, 74], and oleanolic acid derivatives, are widely promoted for their antioxidant and anti-aging effects via NRF2 activation. However, given NRF2’s role in promoting tumor cell survival, therapy resistance, and immune evasion, such interventions may inadvertently create a permissive environment for cancer progression, especially in aging populations where both supplement use and cancer risk are elevated. For example, while resveratrol has been shown to activate NRF2 and enhance cytoprotective gene expression [69], its use in individuals harboring occult or early-stage lung cancers could theoretically contribute to treatment resistance or accelerated tumor growth. These risks are further compounded by the lack of regulatory oversight and biomarker-guided stratification in the use of such supplements. Thus, the therapeutic modulation of NRF2—whether through pharmaceuticals or nutraceuticals—requires careful evaluation of cancer risk, tissue context, and duration of exposure, and should not be universally embraced as a harmless anti-aging strategy. Future clinical trials and public health recommendations should take into account the dual-edged nature of NRF2 biology and ideally integrate NRF2 activity screening into safety assessments of widely consumed NRF2-inducing agents.

Limitations of our study include its retrospective nature and reliance on publicly available microarray data. Although the NRF2 signature was carefully curated and validated, functional studies are needed to confirm causality and identify actionable nodes within the NRF2 network. Prospective validation in contemporary cohorts and across cancer types will further clarify the clinical utility of NRF2-based biomarkers.

In conclusion, NRF2 pathway activation is a strong predictor of adverse outcomes in lung cancer. Our findings underscore the importance of cellular stress resilience pathways at the intersection of aging and cancer biology and open new avenues for biomarker development and therapeutic innovation. Given the widespread, unregulated availability of NRF2-activating nutraceuticals, our findings warrant further safety studies and may inform future regulatory discussions.

## Data Availability

The datasets analyzed in this study are publicly available. Gene expression and clinical data were obtained from the KM Plotter database (http://kmplot.com/analysis/), which integrates datasets from the Gene Expression Omnibus (GEO) repository. Specific GEO accession numbers and dataset processing protocols are detailed in the Methods section. All data used in this analysis can be freely accessed via the original public sources. No new datasets were generated for this study.
